# Individualized structure–function coupling reveals behavioral signatures in the adolescent brain

**DOI:** 10.1162/IMAG.a.1223

**Published:** 2026-04-27

**Authors:** Bahram Jafrasteh, Yangzhi Wang, Qingyu Hu, Keith Jamison, Amy Kuceyeski, Qingyu Zhao

**Affiliations:** Department of Radiology, Weill Cornell Medicine, New York, NY, United States; Tri-Institutional PhD Program in Computational Biology and Medicine, New York, NY, United States; School of Electrical and Computer Engineering, Cornell University, New York, NY, United States

**Keywords:** structure-function coupling, functional connectivity, neuropsychology, adolescent brain, machine learning

## Abstract

Adolescence is a critical period of brain maturation, yet how functional dynamics relate to white-matter microstructure at the individual level remains poorly understood. We developed a machine learning workflow to predict fractional anisotropy (FA) from distributed resting-state functional connectivity (FC) in two large adolescent cohorts: NCANDA (*n* = 814, 12–22 years, longitudinal) and HCP-Development (*n* = 472, 12–22 years, cross-sectional). Whole-brain FA could be modestly predicted from FC (r = 0.16 in NCANDA; r = 0.27 in HCP-D). The accuracy of predicting regional FA significantly varied across 27 white-matter regions, with the highest structure-function coupling detected in fiber tracts subserving unimodal cortical regions. These regional accuracy scores were reproducible between datasets (r = 0.95). Region-specific analyses also revealed tract-clustered FC predictors, highlighting distinct large-scale functional circuits underlying regional microstructural integrity. We then defined a “structure–function gap” as the residual between predicted and observed FA in each white-matter region. These gap measures were significantly associated with a broad constellation of cognitive and behavioral measures, particularly involving memory, impulsivity, and executive function. Notably, significant behavioral coupling emerged in Corticospinal and Cingulum pathways. Together, these findings establish individualized structure–function coupling as a reproducible, anatomically specific, and behaviorally informative marker in youth, offering a new framework to link distributed FC patterns to white-matter development and behavioral variability.

## 1 Introduction

The patterned interactions between neuronal populations give rise to a complex functional architecture, organized within the anatomical scaffold provided by white-matter pathways (Collins et al., 2024; Esfahlani et al., 2022; Suarez et al., 2020). These pathways, composed of myelinated axons, serve as conduits for neural signals, supporting synchronized neural activity between regions (Buianova & Arsalidou, 2021; Lebel & Deoni, 2018). The structural and functional neural systems undergo dynamic changes during adolescence to subserve the development of cognitive, behavioral, emotional, and motor functions (Ladouceur et al., 2012; Park et al., 2022; Sturman & Moghaddam, 2011). Abnormal white-matter microstructural integrity and/or functional co-activity networks are associated with adverse cognitive, emotional, and motor functions, with wide-ranging consequences for neurodevelopmental and neuropsychiatric disorders (Bassett et al., 2018; Bu et al., 2020; McFayden et al., 2024). Thus, understanding the dynamic link between brain structure and function in brain networks is a fundamental pursuit in systems neuroscience.

A growing body of research has sought to characterize the relationship between brain structure and function, often by comparing structural and functional connectivity matrices across individuals or populations (Baum et al., 2020; Collins et al., 2024; Esfahlani et al., 2022; Liu et al., 2022; Suarez et al., 2020; Vázquez-Rodrıguez et al., 2019; Yang et al., 2023). These studies have employed statistical, biophysical, and network communication models to identify associations between the physical architecture of white-matter tracts and patterns of synchronized neural activity. However, most existing approaches are grounded in population-level analyses and focus on average connectivity patterns, leaving open the question of whether individual differences in white-matter microstructural properties can be predicted from functional data. More recent work developed an algorithm called the Krakencoder which focused on mapping structure and function for specific individuals using joint autoencoders with a shared latent space (Jamison et al., 2025), but this framework primarily focuses on global latent alignment rather than explicitly characterizing region-specific structure–function correspondence or deviations. Critically, sparse studies have investigated how structure–function relationships can serve as markers to explain individual differences in cognition, behavior, and mental health.

To address these gaps, we developed a machine-learning workflow to test whether brain functional connectivity (FC) can predict individual differences in white-matter microstructural integrity. This allows us to quantify to what extent the microstructural integrity of individual white-matter fiber tracts constrains or supports large-scale functional organization. Specifically, we selected Fractional Anisotropy (FA) as the primary target metric because it quantifies the directional coherence of water diffusion, serving as a robust proxy for axonal density and myelination (Lebel & Deoni, 2018). These microstructural properties determine the speed and efficiency of signal transmission (Buianova & Arsalidou, 2021), making FA the most direct structural constraint on the synchronized activity patterns measured by FC, particularly during the active white-matter maturation of adolescence. Thus, we posit that the prediction accuracy of FA from FC can serve as a proxy for the statistical dependence between white matter microstructure and FC patterns: high prediction accuracy implies a highly ‘coupled’ regime where functional dynamics are strictly constrained by the structural scaffold, whereas low accuracy suggests a ‘decoupled’ regime where function dissociates from direct anatomical constraints.

Applying such a machine-learning analysis to the longitudinal imaging data from the National Consortium on Alcohol and NeuroDevelopment in Adolescence (NCANDA) (Brown et al., 2015) cohort (*n* = 814, aged 12–22) and an independent validation sample from the HCP Development (HCP-D) (Somerville et al., 2018) study (*n* = 472, aged 12–22), we hypothesized that the strength of structure–function coupling would vary across white-matter regions, and that the functional circuits most predictive of microstructural integrity would differ systematically across major fiber systems. We further hypothesized that the discrepancies between predicted and observed FA, interpreted as structure–function gap, could serve as a biologically meaningful index that represents the degree to which an individual’s white matter architecture supports the normative functional phenotype. These gap measures were then tested as potential markers for individual differences in cognition, personality, and mental health.

## 2 Methods

### 2.1 Participants

We analyzed data from two large adolescent cohorts. The NCANDA dataset consists of 831 adolescents with baseline ages ranging from 12 to 22 years. Out of the 831 participants, we identified subjects who had no structural abnormality at baseline and had both usable resting-state function MRI (rs-fMRI) and Diffusion Tensor Imaging (DTI) available in at least one visit between 12 and 21. This selection resulted in 3201 longitudinal visits from 814 subjects (411 females / 403 males, average age 17.87 years at baseline, maximum 6 longitudinal visits). The Human Connectome Project - Development (HCP-D) (Somerville et al., 2018) dataset contains 652 subjects ages 5 to 22 years. We used 472 subjects ages 12 to 22 years (247 females / 225 males, average age 16.2 years) to match the age range of NCANDA (Table 1).

**Table 1.** Demographic characteristics of participants.

Characteristic	NCANDA (n=814 )	HCP-D (n=472 )
Age at baseline (years)		
Mean (SD)	16.28 (2.37)	16.20 (3.10)
Range	12.0–22.0	12.0–22.0
Sex		
Female	411 (50.5%)	247 (52.3%)
Male	403 (49.5%)	225 (47.7%)
Race/ethnicity		
Caucasian/White	612 (75.2%)	287 (60.8%)
African American/Black	97 (11.9%)	58 (12.3%)
Asian	63 (7.7%)	41 (8.7%)
Other/mixed	42 (5.2%)	86(18.2%)
Socioeconomic status^†^	16.82 (2.50)	-

† Maximum years of education of either parent.

### 2.2 MRI processing

**Fig. 1.**
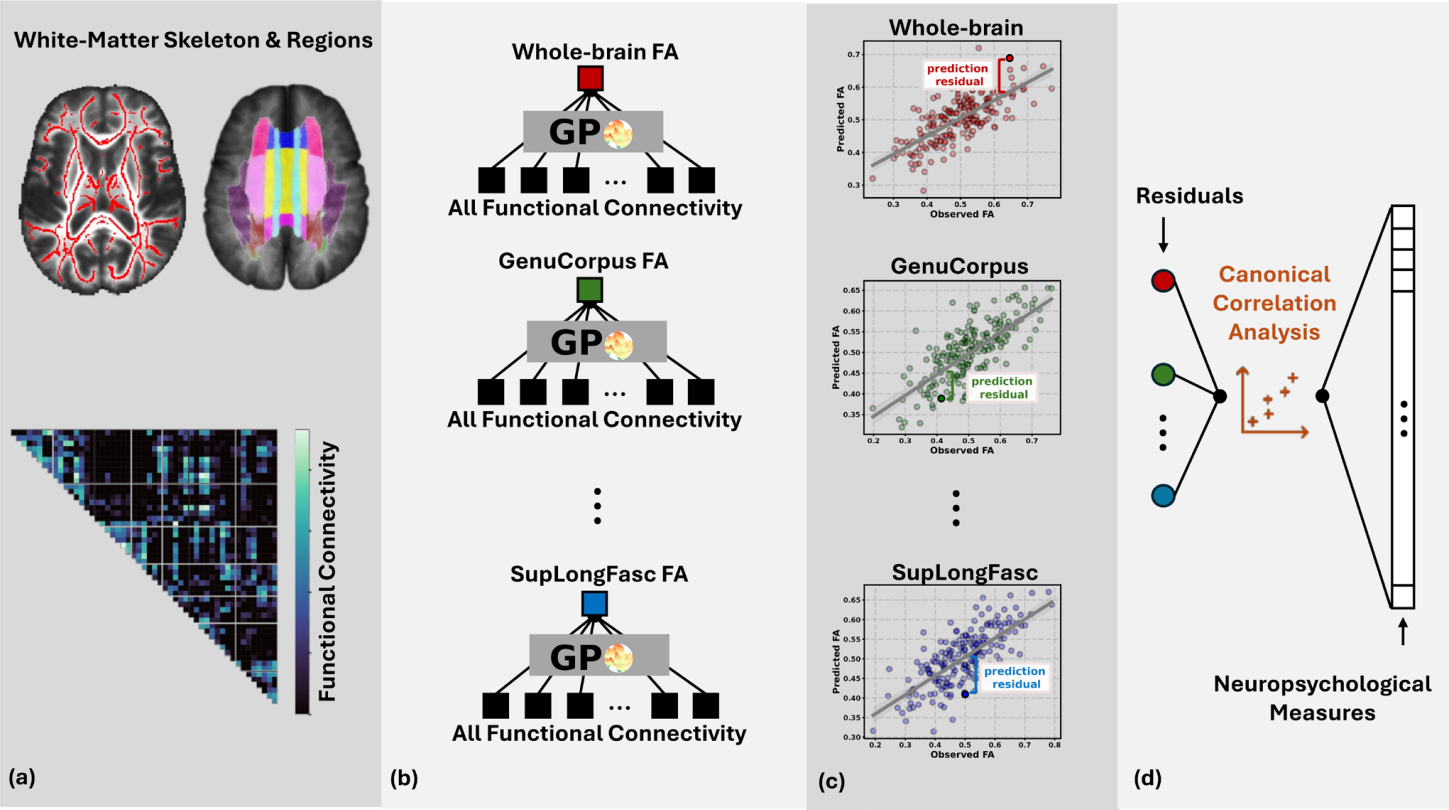
(a) Workflow to map the relationship between resting-state functional connectivity (FC) and white-matter microstructural integrity, quantified by Fractional Anisotropy (FA); (b) Whole-brain FC features were used to predict the FA of the whole-brain white-matter skeleton or the FA within each white-matter region through a Gaussian Process (GP) model; (c) For both whole-brain or regional FA prediction, we computed the residual, the difference between predicted FA by GP and the true FA measured in DTI, for each individual as an index of structure-function gap; (d) Residuals were related to neuropsychological measures by Canonical Correlation Analysis to provide insights into the relationship between structure-function gap and individual variability in behaviors.

To be consistent with prior literature, processing of rs-fMRI and DTI data of NCANDA followed the standard public NCANDA pipeline and our previous works (Zhao et al., 2019, 2021). Rs-fMRI preprocessing included motion correction, outlier-detection, detrending, physiological noise removal, and temporal and spatial smoothing. The pipeline produced the mean blood oxygen level dependent (BOLD) signal from 106 gray matter regions defined by the SRI24 atlas (Rohlfing et al., 2010). Similar to our previous work (Zhao et al., 2025), we computed Pearson’s correlation of BOLD signal between each pair of regions and averaged the correlation values of left and right hemispheres (Fig. 1a bottom, see also Supplementary Methods A), resulting in a 53 ×53 functional connectivity matrix. The preprocessing of DTI followed a previous study (Zhao et al., 2021) that derived the average FA value over the whole-brain tract-based spatial statistics skeleton (Smith et al., 2006), which was further segmented into 27 white-matter regions defined by the JHU-Mori atlas (Mori et al., 2005) (Fig. 1a top).

For HCP-D, all rs-fMRI time series were preprocessed by the HCP minimal preprocessing pipeline, which includes motion correction, susceptibility distortion correction, co-registration to the MNI152 template, and ICA-FIX5 denoising (Glasser et al., 2013; Salimi-Khorshidi et al., 2014). In addition to deriving FC measures based on the SRI24 atlas similar to the NCANDA pipeline, we additionally derived BOLD signal of 86 cortical and subcortical regions defined by the Desikan-Killiany atlas (Desikan et al., 2006) and computed a 43 ×43 connectivity matrix after combining the left and right regions. The DTI data in the HCP-D dataset has been processed to extract FA from the whole brain and 27 white-matter regions from the JHU-Mori Atlas (Mori et al., 2005).

### 2.3 Neuropsychological measures

For the NCANDA cohort, we identified neuropsychological measures following the procedure outlined in Zhao et al. (2025), which included summary scores from 17 test batteries or questionnaires: Penn Computerized Neurobehavioral Test Battery (CNP), Delay Discounting, Grooved Pegboard, UPPS-P Impulsive Behavior Scale, Behavior Rating Inventory of Executive Function (BRIEF), Alcohol Expectancy Questionnaire, Childhood Behavior Check List, Center for Epidemiologic Studies-Depression Scale, Karolinska Sleepiness Scale, Life Experiences Questionnaire, Peer Group Deviance, Response to Stress Questionnaire, Social Support Questionnaire, Ten Item Personality, Rey-O Complex Figure, Paced Auditory Serial Addition Test, and Stroop Test. We removed those measures that were missing for more than half of the participants. As we included participants with heavy drinking behaviors (which was different from Zhao et al., 2025), we added one additional category of behavioral measures related to alcohol use: the number of drinks in the past year, average number of drinks in the past month, and the number of binge episodes in the past month.

### 2.4 Machine learning and statistical analysis

#### 2.4.1 Predicting white-matter integrity from functional connectivity

We first predicted (Fig. 1b) the whole-brain FA value from 1431 FC features, i.e., the upper triangular portion of the FC matrix among 53 bilateral gray-matter regions defined in the SRI24 atlas (Rohlfing et al., 2010) (Fig. 1a bottom). We designed our prediction model as a Gaussian Process (GP) (Jafrasteh et al., 2022; Williams & Rasmussen, 2006) model, which is known to be a non-parametric, probabilistic framework capable of modeling complex non-linear relationships in neuroimaging-based predictive modeling (Marquand et al., 2010; Ortega-Leon et al., 2025). Prediction accuracy was evaluated by 5-fold subject-level cross-validation. In each training run, we fit a regression model to predict each FC and FA measure from potential confounding variables of age, sex, socioeconomic status defined as the maximum years of education of either parent, scanner type, and race/ethnicity by a mixed-effects model with random subject-specific intercepts. Then, the mixed-effects model was applied to all data to remove confounding effects. After testing the model on each subject visit, the predicted FA values of the whole cohort were compared to the observed FA values based on mean squared error (MSE) and Pearson’s correlation r.

To compute the importance of each FC measure for the prediction, we employed a Radial Basis Function kernel with Automatic Relevance Determination (Williams & Rasmussen, 2006) to assign a unique lengthscale parameter to each of the 1431 FC features. A short lengthscale associated with a feature indicates that the GP outcome varies rapidly with the feature, indicating a high relevance for the prediction. Therefore, we defined the feature importance score as the inverse lengthscale (1/l ) for each FC feature, averaged across the 5 cross-validation folds. To compute regional importance scores, we averaged the inverse lengthscale associated with each gray-matter region.

Lastly, beyond the whole-brain FA prediction, we also examined the relationship between whole-brain FC and regional white-matter integrity. To do so, we repeated the machine-learning workflow by replacing the whole-brain FA with the FA value derived from each of the 27 white-matter regions defined by the JHU-Mori atlas (Mori et al., 2005) (see Supplementary Table A1 for full region names).

#### 2.4.2 Clustering FC importance across white-matter regions

To investigate whether white-matter regions belonging to the same fiber tract were predicted by similar FC patterns, we compared the feature importance score of the 1431 FC features across the 27 white-matter regions. First, to ensure the analysis was only based on reliable models, we excluded any region where the observed FA was not a significant predictor of the predicted FA based on a linear mixed-effects model (i.e., p≥0.05 ). The feature importance scores for the remaining white-matter regions were then projected into a two-dimensional space using Uniform Manifold Approximation and Projection (UMAP) (Healy & McInnes, 2024). We then performed a Permutational Multivariate Analysis of Variance (PERMANOVA) test to assess whether the white-matter regions were significantly clustered by the anatomical fiber tract they belong to (Anderson, 2001). All brain surface renderings and white-matter tract visualizations were generated using the Melage visualization tool (Jafrasteh et al., 2023).

#### 2.4.3 Assessing behavioral relevance of the structure-function gap

For each prediction model with a significant prediction accuracy on the NCANDA dataset, we computed the residual, the difference between the predicted and observed FA, as the structure-function gap (Fig. 1c). To link the structure-function gap with individual differences in behavior, we used a regularized Canonical Correlation Analysis (CCA) (Supplementary Methods C) model implemented in the CCA-Zoo (Chapman & Wang, 2021) package and employed a 5-fold subject-level cross-validation. Within each training fold, the regularization parameters (Supplementary Methods C) were optimized via a grid search over 50 logarithmically spaced values between $10^{-4}$ and 1, and mixed-effects models were applied to the neuropsychological measures to regress out the effects from age, sex, race, and socioeconomic status. In addition, to remove the influence of the potential confounding effects from head motion (higher motion might result in worse FA prediction and larger residuals) and magnitude of FA (predicting larger FA might result in larger residuals), we regressed out mean relative displacement (Power et al., 2012) and the observed FA values from these residuals by mixed-effects models. This step was critical to ensure that the resulting gap measures reflected variations in intrinsic network architecture rather than artifacts of scan quality or simple differences in tissue density.

During training, CCA derived a set of canonical components, each represented by a pair of linear transformations of structure-function residuals and the 126 neuropsychological measures (Fig. 1d). The correlation value between the two transformed variables in each component was then computed on the testing fold and averaged over the 5 folds after cross-validation. To identify components with statistical significance, we built a null distribution of correlation values by randomly permuting the neuropsychological measures across subjects. Then, the CCA was recomputed for the permuted dataset. Repeating this process 1000 times resulted in a null distribution of canonical correlation. Finally, the observed correlation was compared against this null distribution to estimate a *p*-value. For a significant component, we computed the canonical loading for each residual and neuropsychological measure to quantify how much they contributed to the canonical correlation. We used the same permutation procedure to identify neuropsychological variables with significantly large loadings.

## 3 Results

### 3.1 Estimating whole-brain FA from functional connectivity

**Fig. 2.**
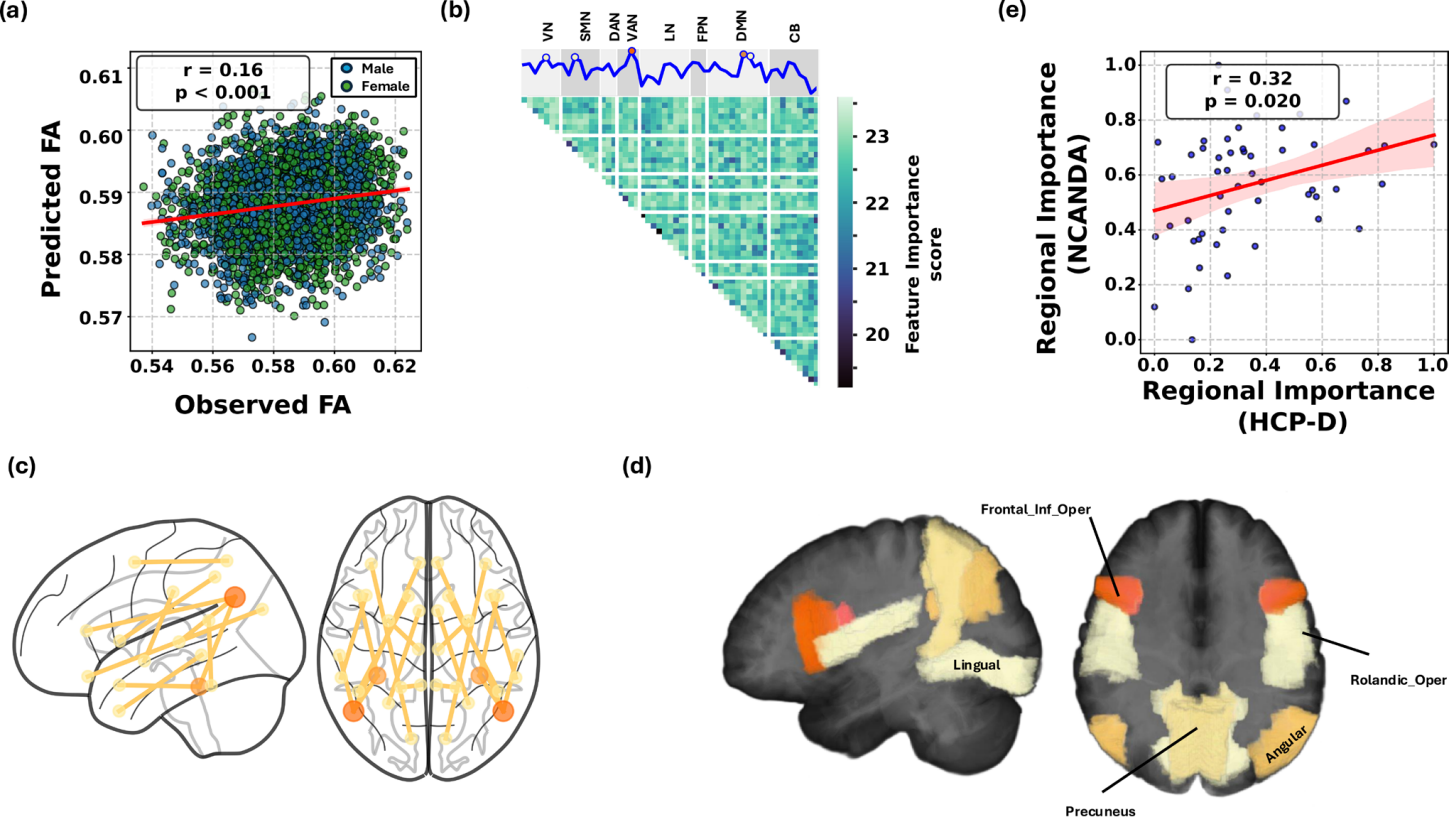
(a) Cross-validating the GP model on NCANDA data revealed that the whole-brain FA predicted by FC significantly correlated with the observed FA from DTI; (b) Average feature importance score (inverse lengthscales) from the GP model, to quantify how each FC feature contributed the prediction of FA. The sum of importance scores associated with each of the 53 gray-matter regions quantified the regional importance score for FA prediction; (c) Top 10 FC features contributing the most to FA prediction; (d) Top 5 gray-matter regions contributing the most to FA prediction; (e) The regional importance scores for NCANDA significantly correlated with those derived from the HCP-D study.

Using a GP model to predict the whole-brain FA from 1431 FC features among 53 bilateral gray-matter regions, a 5-fold subject-level cross-validation revealed that the prediction achieved an MSE of 0.0003 and a r = 0.16 (p<0.001 ) between predicted and observed FA values (Fig. 2a). The prediction of whole-brain FA was less accurate when using FC among 106 unilateral gray-matter regions as the input to GP model (r = 0.11, Supplementary Fig. A1a), suggesting that bilateral averaging of FC improved predictive performance. To explicitly assess the impact of scanner difference on predictive performance, we performed a stratified sensitivity analysis based on scanner type. Without retraining the model, significant prediction accuracy was observed for both scanner types (GE: r=0.12 , $p<10^{-8}$ , Siemens: r=0.25 , $p<10^{-15}$ ). To assess whether the prediction model was invariant to sex, we performed cross-sex generalization analyses. A model trained exclusively on female participants successfully predicted FA in male participants (r=0.15 , p<0.001 ), and conversely, a model trained on male participants successfully predicted FA in female participants (r=0.17 , p<0.001 ).

To understand which FC features were used for prediction, we defined feature importance score as the inverse lengthscales from the GP model (See Section 2.4). As the coefficient of variation (Abdi, 2010) of the importance scores of whole-brain FA prediction was relatively low across the 5 folds (< 2%, Supplementary Table A2), Figure 2b visualizes the importance scores averaged over the folds, reshaped into the upper triangular portion of the connectivity matrix, with brighter entries showing the critical FC that drove the FA prediction. The top 10 FC with the highest importance are displayed in Figure 2c, which highlighted the FC among Angular Gyrus, Inferior Frontal Lobe, Fusiform Gyrus, and other Temporal Lobe regions. Next, to get insights on which brain regions were associated with more important FC for whole-brain FA prediction, Figure 2b further visualizes the regional importance as the average value of feature importance scores associated with each of the 53 gray-matter regions, which were grouped into 8 broader functional networks (Supplementary Table A3). Gray-matter regions with highest importance included the Inferior Frontal Gyrus (Opercular part), Angular Gyrus, and Precuneus (Fig. 2d). Furthermore, the regional feature importance scores derived from the male-only and female-only models were significantly correlated (r=0.30 , p=0.03 ; Supplementary Fig. A2). These results suggest that the FC-FA mapping generalized well across sexes and was driven by anatomically similar features.

Lastly, we conducted two replication analyses on the external HCP-D dataset to confirm the generalizability of findings. First, we trained the model on the NCANDA cohort and applied it to the HCP-D dataset, which yielded a significant prediction accuracy (r=0.10 , p=0.03 ). Next, we conducted a separate 5-fold cross-validation within the HCP-D dataset by training GP models using the Desikan-Killiany atlas and compared the regional importance scores between the two atlases from the two datasets. Specifically, the cross-validated model achieved an MSE of $5.9\times 10^{-5}$ , and an r = 0.27 (Supplementary Fig. A1b) on HCP-D, and the regional importance derived from the two datasets significantly correlated (r = 0.32, p=0.02 , Figure 2e, see Supplementary Methods D for the procedure of transferring regional importance scores across the two atlases).

### 3.2 Estimating FA in white-matter regions

**Fig. 3.**
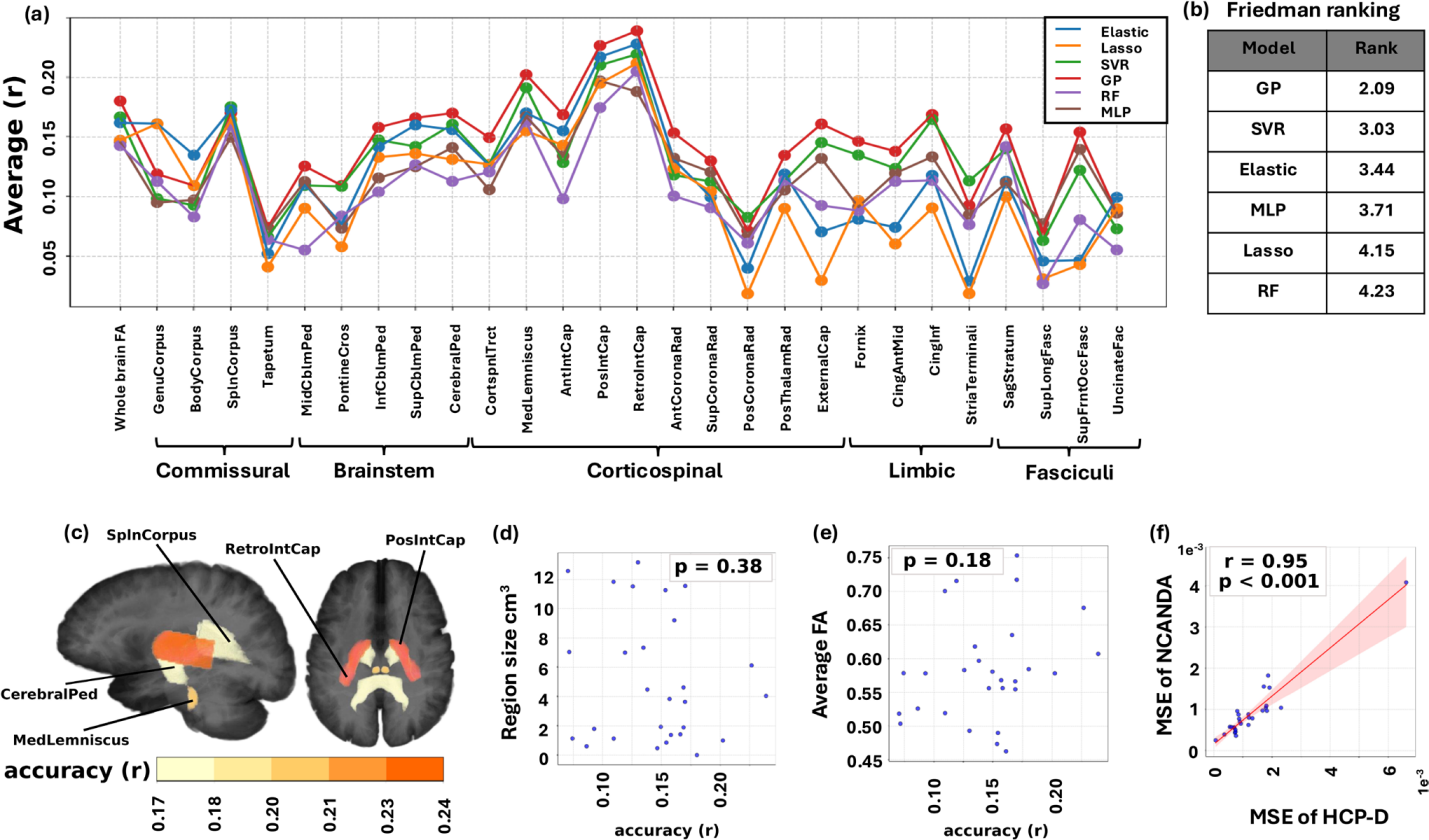
(a) Average prediction accuracy (r) over 5-fold cross-validation of Gaussian Processes (GPs) and other machine-learning models for predicting whole-brain FA and regional FA in 27 white-matter regions in NCANDA (Supplementary Table A1); (b) GPs achieved the best prediction performance based on Friedman ranking; (c) Top 5 white-matter regions with highest prediction accuracy; (d, e) The prediction accuracy did not significantly correlate with the size of the white-matter region or the magnitude of FA; (f) Accuracy of FA prediction in the 27 white-matter regions significantly correlated between NCANDA and HCP-D.

Based on the same machine-learning workflow, we predicted the FA within each white-matter region based on whole-brain FC. Results suggest that the accuracy of FA prediction substantially varied across white-matter regions (Fig. 3a). While the magnitude of accuracy was modest, a robust relative hierarchy emerged: the highest accuracy (Fig. 3c) was recorded for the Retrolenticular Part of Internal Capsule (r = 0.24), Posterior Limb of Internal Capsule (r = 0.23), and Medial Lemniscus (r = 0.20). On the other hand, the prediction in Superior Longitudinal Fasciculus (r = 0.07) was the least accurate. To ensure that such accuracy variation truly reflected the difference in regional structure-function relationships and was not biased by our specific choice of machine-learning models, we repeated the analysis by replacing GP model with Multi-Layer Perceptron (MLP), Lasso, Random Forest (RF), Elastic Net (Elastic) and Support Vector Regression (SVR) (Supplementary Methods B). Figure 3a illustrates that all machine-learning models resulted in the same trend of accuracy variation across regions, despite that GP was in general more accurate than other models according to the Friedman ranking (Demšar, 2006) (Fig. 3b).

Furthermore, we examined whether the relative ranking of prediction accuracy was biased by the size of the white-matter region (${\text{in cm}}^{3}$) or the strength of fiber integrity itself. To investigate this, regional r was correlated with white-matter region volume and with regional FA averaged over all subjects, both resulting in insignificant correlations (p=0.38 and p=0.18 , Fig. 3d, 3e). Lastly, we repeated the regional FA prediction in the 27 white-matter regions on the HCP-D dataset. Based on the GP model, Figure 3f shows that the regional prediction accuracy obtained from NCANDA significantly correlated with that from the HCP-D dataset (r = 0.95, p<0.001 ), confirming that this regional variance of predictability was stable.

### 3.3 Distinct functional circuits driving regional FA prediction

**Fig. 4.**
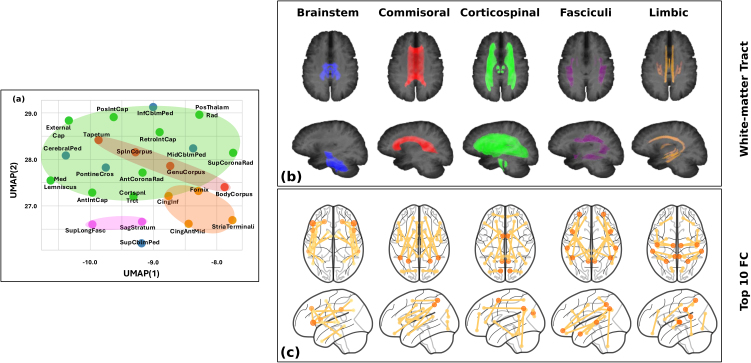
(a) 2D UMAP visualization of feature importance scores associated with 24 white-matter regions indicated that regions belonging to the same fiber tract significantly clustered together (p<0.03 , PERMANOVA); (b) Visualization of 5 major fiber tracts; (c) Top 10 FC features contributing the most to FA prediction in Brainstem, Commissural, Corticospinal, Fasciculi, and Limbic Tracts.

We next examined whether the functional circuits predicting microstructural integrity were organized by anatomical white-matter tracts. To ensure this analysis was based on reliable models, we excluded the Posterior Corona Radiata, Superior Longitudinal Fasciculus, and Uncinate Fasciculus, where the observed FA was not a significant predictor of the predicted FA based on linear mixed-effect models (p>0.05 ). The subsequent analysis was performed on the remaining 24 white-matter regions. A visualization of their feature importance scores (inverse lengthscales) using UMAP revealed a distinct clustering pattern (Fig. 4a). A subsequent PERMANOVA test confirmed that the white-matter regions belonging to the same fiber tract were predicted by significantly similar FC patterns, which were distinct from the patterns of other tracts (p<0.03 ). This clustering pattern endured even when including the models for all 27 white-matter regions (p<0.01 , Supplementary Fig. A3).

To get insights on which specific FC drove regional FA prediction, we further averaged feature importance scores over regions within the same white-matter tract and displayed top 10 FC that contributed the most to the prediction of FA in each tract (Fig. 4b, c). The results suggest that the microstructural integrity of the Brainstem tract was primarily predicted by functional connections to the Frontal Lobe. The FA of the Commissural tract was mainly predicted by connectivity between Parietal (both Inferior and Superior) regions and Subcortical Nuclei (e.g., Thalamus and Caudate). The FA of the Corticospinal Tract was mainly predicted by functional connections to the Precuneus and Supplementary Motor Area. For the Limbic tract, key predictive connections involved Supramarginal Gyrus, Posterior Cingulum, Superior Temporal Lobe, and Superior Parietal Lobe. Finally, the Fasciculi tract was best predicted by connections linking Inferior Parietal Lobe to Temporal Lobe and Orbital Middle Frontal Gyrus.

### 3.4 Structure-function gap was linked to individual differences in neuropsychological behaviors

**Fig. 5.**
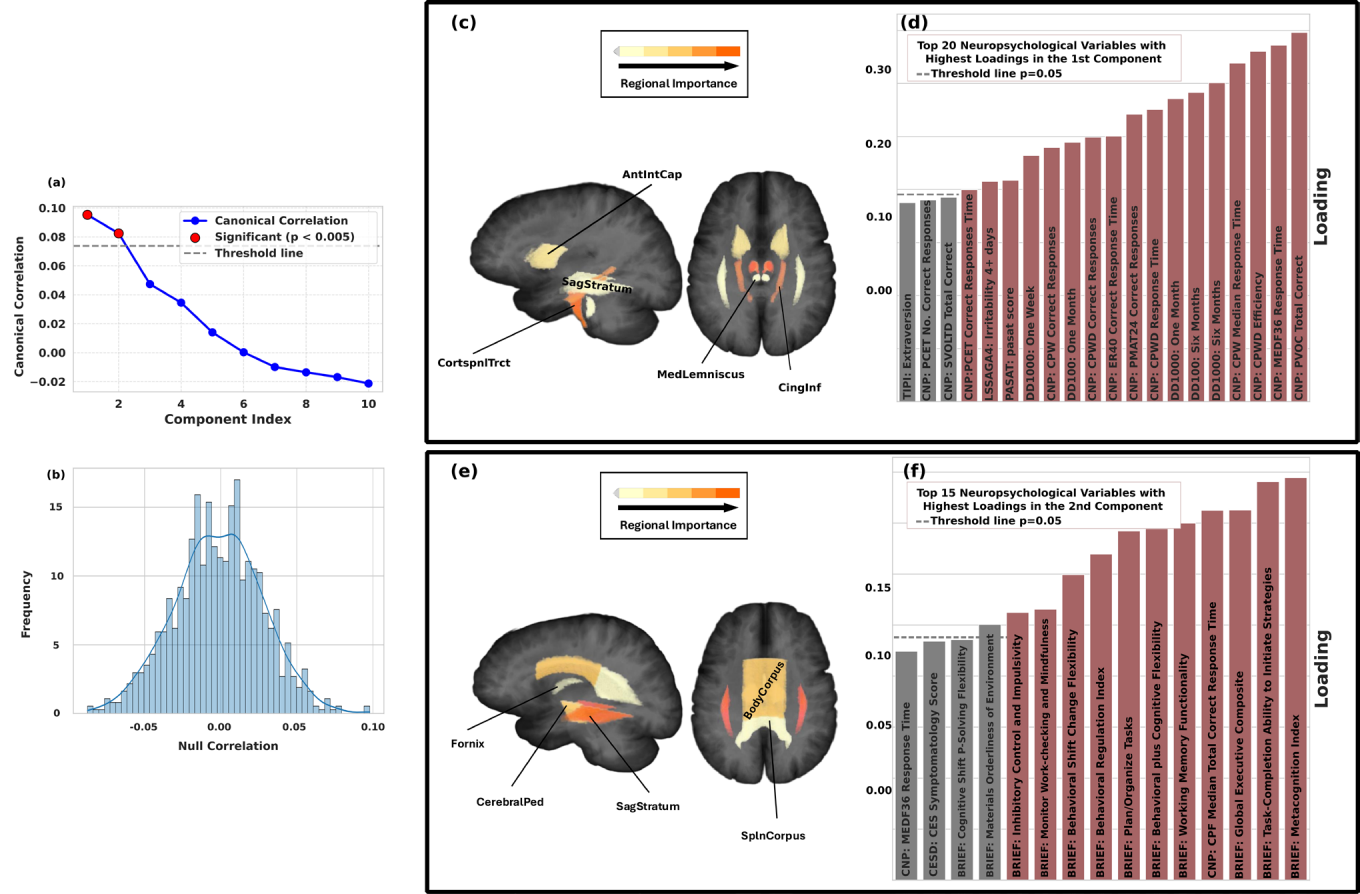
(a) Canonical correlations of the top 10 components that linked 25 residuals for FA prediction to 126 neuropsychological measures. The canonical correlation of the first two components were statistically significant based on permutation test (p<0.005 , gray dashed line); (b) Null distribution of correlation obtained from the permutation test; (c) Top five white-matter regions whose residuals with largest canonical loadings in the first canonical component; (d) Neuropsychological measures with significantly large canonical loadings in the first canonical component (p<0.05 ). (e, f) White-matter regions and neuropsychological measures defining the second canonical component. CNP: Penn Computerized Neurobehavioral Test Battery; PASAT: Paced Auditory Serial Addition Test; TIPI: Ten Item Personality; DD: Delay Discounting; CESD: Center for Epidemiologic Studies-Depression Scale; BRIEF: Behavior Rating Inventory of Executive Function.

The discrepancy between the predicted FA by GPs and the observed FA measured via DTI was used as an index of structure-function gap. Specifically, for the whole-brain FA prediction and each regional FA prediction, we computed the difference between predicted and actual FA at each participant visit (Fig. 1c), resulting in 25 residual values (24 regions with accurate FA prediction, 1 whole-brain) corrected for head motion and magnitude of FA. We examined whether the residuals could explain individual differences in behavior and cognition. We used CCA to relate the 25 residuals to 126 neuropsychological measures (Zhao et al., 2025) from 17 neuropsychological test batteries or behavioral questionnaires that assessed socioemotional status, cognitive control functions, and substance use. A subject-level 5-fold cross-validation of the CCA model revealed that the two leading canonical components highlighted in Figure 5a had significant canonical correlations of 0.10 and 0.08 (p<0.001 , Fig. 5b, Supplementary Fig. A4). These correlations were higher than those derived by a CCA relating the neuropsychological measures to traditional coupling metrics based on the Spearman’s correlation between regional structural connectivity (SC) and FC profiles (Supplementary Methods E).

Next, to understand which constellation of residuals and neuropsychological measures were coupled, we examined the magnitude of canonical loadings of the variables and used a permutation test to identify those variables with statistically high loadings (p<0.05 ) (Supplementary Fig. A5). The top 5 contributing regions for the first component were the Corticospinal Tract, Inferior Cingulum, Anterior Limb of Internal Capsule, Sagittal Stratum, and Medial Lemniscus (Fig. 5c). Across these regions, greater structure–function mismatch, reflected by lower white-matter integrity relative to FC-predicted levels, was associated with poorer performance in episodic memory (e.g., visual object learning and word memory, PASAT), fluid reasoning (e.g., Penn Matrix Reasoning), Emotion recognition (e.g., Penn Emotion Differentiation), and longer delay discounting (Fig. 5d, see the direction of loadings in Supplementary Fig. A6a, c). The second significant component was characterized by strong loadings in the Sagittal Stratum, Cerebral Peduncle, Body of Corpus Callosum, Fornix, and Splenium of Corpus Callosum (Fig. 5e). Higher structure–function mismatch in these regions was linked to worse scores in 11 behavioral variables, almost exclusively from the BRIEF questionnaire, including scores for task monitoring, working memory, planning, and inhibition (Fig. 5f, Supplementary Fig. A6b, d). Identified neuropsychological measures in the two components largely overlapped with those identified by the CCA based on traditional SC-FC coupling metrics (Supplementary Fig. A7, Supplementary Table A4).

## 4 Discussion

Based on a machine-learning model applied to two independent large-scale multi-modal neuroimaging studies of adolescents (12–22 years old), this study provided evidence that resting-state FC could serve as a modest yet significant predictor of individual differences in white-matter microstructure during adolescence. The predictive patterns were found to be anatomically specific and behaviorally informative (Fig. 2). Specifically, regional FA predictions showed substantial variability in prediction accuracy across 27 white-matter regions, with comparatively high predictability observed in the Corticospinal Tracts, including the Retrolenticular Part of Internal Capsule, Posterior Limb of Internal Capsule, and Medial Lemniscus (Fig. 3). Functional circuits that drove the prediction differed across major white-matter pathways and were not confined to local anatomical neighborhoods (Fig. 4). Individual differences in the gap were linked to specific cognitive and behavioral profiles. We identified two significant components: one linking the gap in the Corticospinal Tract and Inferior Cingulum to high-order cognitive functions measured in CNP and impulsivity measured by Delay Discounting, and a second linking the gap in tracts like the Sagittal Stratum to broad executive functions measured in BRIEF (Fig. 5). These findings underscore the value of machine-learning models in revealing individualized, anatomically specific, and behaviorally relevant relationships between brain structure and function.

A growing number of studies have used stylized network models of communication to associate brain function and structure (Baum et al., 2020; Fotiadis et al., 2024; Liu et al., 2022; Vázquez-Rodrıguez et al., 2019; Yang et al., 2023). These studies have largely relied on statistical associations or group-level regressions to characterize how structural and functional connectivity co-vary across regions or individuals. For example, a traditional approach defines structure–function coupling as the Spearman’s correlation between a region’s SC profile, typically derived from tractography-based streamline counts (Maier-Hein et al., 2017; Schilling et al., 2018), and its FC to other regions (Baum et al., 2020; Gu et al., 2021; Vázquez-Rodrıguez et al., 2019). One limitation of this definition is that tractography often tends to reconstruct short-range connections (Maier-Hein et al., 2017; Sotiropoulos & Zalesky, 2017) capturing local, direct physical pathways, whereas FC reflects distributed, long-range synchronization that may arise through indirect pathways or network-level dynamics. Under this conceptual mismatch, a rank-based correlation between SC and FC profiles conflates fundamentally different biological processes and thus offer only a partial characterization of structure–function relationships. In contrast, our framework addresses a different question: to what extent does the microstructural integrity of a single white-matter fiber tract constrain or support large-scale functional organization? By relating whole-brain FC to tract-specific FA, we move beyond node-wise structure–function correspondence and instead model the distributed functional footprint of individual fiber bundles.

Besides the way to quantify structure-function relationships, another open question is whether the brain’s functional organization can be predicted by structural properties (or vice versa) on the individual level, because in many individual-level predictive frameworks, group average FC was an equivalent or better predictor of individual FC than the SC-based predictive models (Smolders et al., 2024). More recent machine-learning analyses have focused on improving individual-level identifiability in translating whole-brain structural or functional connectivity matrices (Chen et al., 2024; Wang et al., 2022; Zhang et al., 2022). For example, Krakencoder (Jamison et al., 2025), a unified connectome translation model that jointly maps between SC and FC and across atlases via a shared latent space, achieved substantially higher individual-level identifiability than prior structure-function association models. However, such matrix-level predictions primarily reflect global similarity between the whole connectome metrics and typically do not yield tract-specific or circuit-specific insights. By contrast, our model can identify which specific functional circuits were associated with an individual white-matter tract and is capable of generating region-specific structure-function gap.

Prior studies have shown that structure–function relationships vary systematically across the neocortex (Baum et al., 2020; Gu et al., 2021; Valk et al., 2022; Vázquez-Rodrıguez et al., 2019; Yang et al., 2023), with stronger coupling typically observed in unimodal regions such as primary sensory and motor cortices, where local anatomical connections tightly constrain functional dynamics. In contrast, transmodal areas, including those within the default mode network, often exhibit weaker coupling, likely due to their integrative roles and more distributed connectivity patterns. Consistent with this spatial heterogeneity, our results also demonstrate substantial variability in the predictability of regional FA across white-matter tracts. In line with this traditional gradient-based coupling hierarchy, our findings show that the highest predictability was not in transmodal association tracts, but rather in unimodal projection and sensory pathways. Specifically, the Posterior Limb of the Internal Capsule and the Medial Lemniscus contain somatosensory fibers subserving sensory and motor signals, while the Retrolenticular Internal Capsule connects to the primary visual cortex (Behrens et al., 2003). In these regions, functional dynamics are likely more tightly constrained by local anatomical integrity. In contrast, transmodal association tracts like Superior Longitudinal Fasciculus showed lower coupling, aligning with their established role in supporting more distributed and flexible connectivity patterns (Mesulam, 1998; Thiebaut de Schotten et al., 2011). These findings reinforce the principle that structure-function coupling is relatively stronger in unimodal systems, and suggest that whole-brain resting-state FC patterns are most reflective of microstructural integrity in these fundamental sensorimotor pathways. However, we note that the absolute magnitude of these associations remains modest, likely reflecting both the inherent noise in FC and the limitations of FA as a proxy for microstructure in regions with complex crossing fibers.

A key advantage of predicting regional white-matter microstructure from whole-brain functional connectivity is the ability to uncover non-local, distributed functional circuits that are supported by specific white-matter fiber bundles. The observed structure–function coupling patterns largely align with known anatomical and functional relationships. For example, the superior longitudinal and Arcuate Fasciculi are known to course through the Inferior Parietal Lobule to connect temporal and frontal regions (Basile et al., 2024; Janelle et al., 2022; Kamali et al., 2013), both pathways reflected in their associated FC patterns. Similarly, the Corticospinal Tract supports sensorimotor integration and fine motor control (Moreno-López et al., 2016), with its functional relevance anchored in Supplementary Motor Area, Precuneus, and other related regions in the Superior Parietal Lobe. However, structure-function coupling might not be restricted to spatially adjacent regions, such as the strong association between frontal FC and the integrity of Brainstem fiber tracts, which indicates the frontal network integration may depend on long-range pathways traversing through the Brainstem. This challenges the traditional view that structure–function coupling is primarily local, and instead supports a model in which local white-matter microstructure influences the organization of large-scale, integrative functional networks.

Recent studies have also suggested that structure–function coupling is associated with individual variability in cognition and behavior (Baum et al., 2020; Gu et al., 2021; Popp et al., 2024). For instance, stronger coupling in the right anterior insula/putamen and weaker coupling in the middle cingulate/supplementary motor areas both predicted higher composite cognitive scores (Gu et al., 2021), and structure–function coupling in the rostrolateral prefrontal cortex was associated with executive functioning (Baum et al., 2020). However, these studies typically investigated only a single composite score and lacked comprehensive coverage of diverse behavioral domains. In contrast, our study significantly extends this line of inquiry by examining the structure–function gap in relation to a broad set of 126 neuropsychological measures, revealing two significant behavioral components. The first component highlighted a coupling between higher-order cognitive functions (memory and social cognition) and impulsivity (delay discounting) with the structure–function gap in the corticospinal tract and inferior cingulum. Specifically, greater structure–function mismatch, characterized by lower white-matter integrity relative to FC-predicted levels, was associated with poorer performance in memory- and social cognition–related measures, alongside lower delay discounting. This finding aligns with current understanding that the inferior (hippocampal) cingulum provides a clear substrate for memory, while the corticospinal tract may reflect motor or response-related aspects of task performance (Lemon, 2008; Rolls, 2019). The second component was linked to broad executive function skills, particularly meta-cognition and task completion ability, and was underpinned by the cerebral peduncle, a key component of cortico-subcortical motor pathways, and the corpus callosum, the primary conduit for interhemispheric communication. For this component, greater structure–function mismatch was consistently associated with worse executive functioning, as reflected by higher BRIEF scores. Together, these findings suggest that different cognitive domains exhibit distinct sensitivity to the direction and magnitude of structure–function mismatch, with memory and emotion recognition particularly vulnerable to insufficient structural support for functional engagement, and executive control dependent on precise alignment across distributed integrative pathways.

### 4.1 Limitations

Although we demonstrated that averaging bilateral functional connectivity preserved sensitivity in predicting FA, this approach might obscure lateralized structure–function relationships. For example, cross-hemispheric functional connectivity supported by Callosal white-matter pathways might not be fully captured. It is worth noting that our choice of parcellation was guided by the standard processing pipeline released by the NCANDA study, which only contains 40 bilateral cortical regions. Although this choice ensures consistency with prior NCANDA analyses, it necessarily limits spatial resolution. A systematic evaluation of how alternative parcellation schemes, particularly higher-resolution atlases (e.g., Schaefer-400 (Schaefer et al., 2018)), affect the prediction accuracy remains an important direction for future work.

Another limitation of this study is that the structure–function gap used in the brain–behavior analyses was derived from FA prediction models trained within the same cohort, introducing a small degree of dependence between model estimation and downstream analysis. Although we considered training FA prediction models in HCP-D to generate fully independent gap measures in NCANDA, such models did not generalize reliably due to limited sample size and cross-sectional design of HCP-D. Future studies with larger, harmonized datasets will enable fully independent training and validation of structure–function gap measures.

Lastly, our prediction models only achieved modest accuracy (r = 0.2 - 0.4). One reason might be that we did not account for the influence of genetic variation or fine-grained environmental factors in shaping individual differences in brain connectivity and microstructure, which contributed to reduced effect sizes by increasing population heterogeneity.

In conclusion, this study established a robust machine-learning framework for mapping individual-level structure-function coupling in the developing adolescent brain. By validating our models across two large-scale independent cohorts, we demonstrated that resting-state FC can reliably predict white-matter microstructural integrity, with the highest accuracy observed in fundamental sensorimotor pathways. Crucially, we identified the “structure-function gap”, the deviation between predicted and observed integrity, as a biologically meaningful marker of individual variability rather than mere prediction error. This gap captured complex behavioral associations with impulsivity, memory, and executive function. Together, these findings challenge strictly local models of structure-function coupling and offer a novel, individualized approach for characterizing the heterogeneous trajectories of adolescent neurodevelopment, supporting personalized risk stratification, and informing early identification and monitoring of neurodevelopmental vulnerability.

## Supplementary Material

Supplementary Material

## Data Availability

The data that support the findings of this study were sourced from two publicly available datasets. Data from the National Consortium on Alcohol and NeuroDevelopment in Adolescence (NCANDA) were based on a formal, locked data release NCANDA-NIAAADA-BASE-V01, NCANDA-NIAAADA-01Y-V01 to NCANDA-NIAAADA-09Y-V01 available via https://nda.nih.gov/edit_collection.html?id=4513. Data from the Human Connectome Project - Development (HCP-D) are available from the NIMH Data Archive (NDA) at https://www.humanconnectome.org/. The code is available upon request.
