# Supplementary material for: Individualized structure–function coupling reveals behavioral signatures in the adolescent brain
Source: Imaging Neurosci (Camb). 2026 Apr 27;4:IMAG.a.1223. doi: 10.1162/IMAG.a.1223 (PMC13125062; doi:10.1162/IMAG.a.1223)
Supplement: Supplementary Material [file IMAG.a.1223_supp.pdf]

Supplementary Material

A Functional Connectivity Measurement

To extract functional connectivity information from the NCANDA dataset, we excluded 3 cerebellar vermis regions from the 109-region parcellation (available at https://www.nitrc.org/projects/sri24). The remaining 106 regions were then paired bilaterally to form a total of 53 regions. Using the processed dataset, we constructed a 53×53 correlation matrix to capture functional connectivity patterns:

- **Diagonal Elements:** For each bilateral region, we calculated the correlation between the left and right regions as the FC in the diagonal of the matrix.
- **Off-Diagonal Elements (Upper Triangle):** For each pair of bilateral regions, the FC was the average of:
  - **Left-Left (LL):** Correlation between the two regions in the left hemisphere.
  - **Right-Right (RR):** Correlation between the two regions in the right hemisphere.
  - **Left-Right (LR):** Correlation between the first region in the left hemisphere and the second region in the right hemisphere.
  - **Right-Left (RL):** Correlation between the first region in the right hemisphere and the second region in the left hemisphere.

This method resulted in a symmetric 53×53 FC matrix. We used the upper triangular portion of the matrix for FA prediction.

B Regression Models for Predicting FA Values

We performed regression analysis to predict FA values for each white-matter (WM) region. Several machine learning models were evaluated using 5-fold cross-validation (CV) to assess their performance. All models were implemented using `sklearn`, except for the Multi-Layer Perceptron (MLP) and Gaussian process (GP), which was implemented using PyTorch. Below is a detailed description of each model used:

- **Multi-Layer Perceptron (MLP):** The MLP model was configured with one hidden layer consisting of 50 hidden nodes. The model was trained for 100 epochs with a batch size of 64. Early stopping was applied to prevent overfitting, halting training once the model's performance on the validation set ceased to improve after a specified number of epochs.

- **Lasso Regression:** Lasso regression is a linear model with L1 regularization, which helps in feature selection by shrinking some coefficients to zero. This property is particularly useful when there are many irrelevant or redundant features in the dataset, as it improves model interpretability and reduces overfitting.

- **Gaussian Process Regression (GP):** Gaussian Process (GP) Regression provides a probabilistic, non-parametric framework for regression tasks, offering both predictions and uncertainty estimates. We used a Radial Basis Function (RBF) kernel with Automatic Relevance Determination (ARD). The ARD mechanism assigns a unique lengthscale hyperparameter to each input feature (i.e., each functional connection). This allowed us to quantify feature importance score, defined as the inverse lengthscale, as described in the main text (Section **??**). GP is particularly well-suited for modeling complex, non-linear data patterns, and it was selected as the primary model for all subsequent analyses after demonstrating superior predictive performance compared to other models.

- **Elastic Net:** Among the linear models, Elastic Net stands out due to its balance of L1 (Lasso) and L2 (Ridge) regularization. This combination makes it effective for handling datasets with highly correlated features, allowing both feature selection and regularization. For optimal performance, within each fold of the cross-validation, the α parameter was selected from {1e-2, 2e-2, 5e-2, 1e-1, 2e-1, 0.5, 1} and the l_1 ratio from {0.1, 0.5, 0.7, 0.9, 0.95, 0.99, 1}.

- **Support Vector Regression (SVR):** Support Vector Regression, based on Support Vector Machine principles, was used with an RBF kernel to capture non-linear relationships. SVR is robust in high-dimensional spaces and is effective for modeling complex patterns between input functional connectivity features and the target FA values.

- **Random Forest Regression (RF):** Random Forest is an ensemble learning method that constructs multiple decision trees and combines their predictions to improve accuracy. Here, we used 500 trees.

C Canonical Correlation Analysis

Canonical Correlation Analysis (CCA) is a statistical technique used to explore the relationships between two sets of variables. Formally, we suppose we have two sets of measurements X, Y from n samples, where X is a $p \times n$ matrix, and Y is a $q \times n$ matrix. CCA forms linear transformations a and b such that $U = aX$ and $V = bY$, where U and V are called the Canonical Variates of the n samples. CCA finds a and b such that the correlation between U and V is maximized: $r = corr(U, V)$.
To make the analysis more robust and stable, we used a Regularized Canonical Correlation Analysis (rCCA), specifically using L_2 (Ridge) regularization. This method adds a penalty term to the optimization, which helps to stabilize the covariance matrices and prevent the canonical weights (a and b) from becoming excessively large. This is controlled by two regularization parameters, c_1 and c_2, which apply penalties to the X variables (structure-function gap residuals) and Y variables (neuropsychological measures), respectively. As described in the main text, these parameters were optimized via grid search within each cross-validation fold to find the values that maximized the canonical correlation on held-out data.

The first canonical variate pair (U_1, V_1) captures the strongest relationship between the two datasets. Subsequent canonical pairs, or components, (U_2, V_2), (U_3, V_3), etc. are computed sequentially, subject to being uncorrelated with previous pairs. Each pair represents a new dimension of brain-behavior association. Canonical Correlation r_i measures the strength of association between the two canonical variables of the ith component. Canonical Loading of variable j (the jth residual measure or the jth neuropsychological measure) for component i is defined as the correlation between the original j^{th} variable in X (or Y) and the ith canonical variable, i.e., $loading(X_{(i,j)}) = corr(x_j, U_i)$ and $loading(Y_{(i,j)}) = corr(y_j, V_i)$. A high loading $X_{(i,j)}$ (or $Y_{(i,j)}$) indicates that the jth residual (or neuropsychological) measure plays a major role in defining the ith canonical component. Because the sign of a canonical component is arbitrary, we evaluated the strength of the association by computing the absolute value of the canonical correlation coefficient across testing folds. To do so, we assessed sign consistency across testing folds by computing correlations between component loadings. When an opposite sign was detected, the corresponding canonical variates were inverted to ensure consistent orientation across folds. Lastly, the absolute values of the loadings were ranked across brain and neuropsychological variables.

D Cross-Atlas Mapping via Overlap Matrix

To validate the robustness of our feature importance findings across independent cohorts, we compared the predictive models trained on the NCANDA dataset (using the SRI24 atlas, 53 regions) with those trained on the HCP-D dataset (using the Desikan-Killiany (DK) atlas, 43 regions). Since the feature importance vectors differ in dimensionality (53×53 vs. 43×43) and anatomical definition, a direct element-wise correlation was impossible. We therefore developed a Spatial Overlap Matrix (M) to mathematically project the feature weights from the source space (Desikan-Killiany) into the target space (SRI24).

Both atlases were first registered to a common standard space (MNI152, 2mm resolution). We defined a transformation matrix $M \in \mathbb{R}^{N_{SRI} \times N_{DK}}$, where $N_{SRI} = 53$ and $N_{DK} = 43$. Each element M_{ij} represents the spatial contribution of the j-th region in the Desikan-Killiany atlas to the i-th region in the SRI24 atlas. This was calculated via voxel-wise intersection:

$$M_{ij} = \frac{|V_i^{SRI} \cap V_j^{DK}|}{|V_i^{SRI}|}$$

where:V_i^{SRI} is the set of voxels belonging to region i in the SRI24 atlas. V_j^{DK} is the set of voxels belonging to region j in the Desikan-Killiany atlas. $|\cdot|$ denotes the volume (count of voxels). This normalization ensures that each row of M sums to approximately 1 (accounting for minor spatial mismatches), effectively representing each SRI24 region as a weighted linear combination of Desikan-Killiany regions.

Let $\mathbf{W}_{DK}$ be the 43×43 symmetric matrix of feature importance scores derived from the HCP-D models, representing the predictive weight of functional connections between DK regions. To map this matrix into the SRI24 geometry, we applied a bilateral linear projection:

$$\mathbf{W}_{Projected} = M \cdot \mathbf{W}_{DK} \cdot M^{\top}$$

The resulting matrix $\mathbf{W}_{Projected}$ was a 53×53 matrix where the importance of a connection between SRI regions i and k was estimated as the weighted sum of the importance scores of their constituent DK regions.

E CCA Based on Traditional SC-FC Coupling Metrics

E.1 Calculation of Traditional SC-FC Coupling Metrics

We defined traditional structure-function coupling using the "node-centric" approach (Baum et al., 2020; Gu et al., 2021; Vázquez-Rodríguez et al., 2019). For each participant, we computed the structural connectivity (SC) and functional connectivity (FC) profiles for 40 bilateral gray-matter regions defined in the SRI24 atlas. The SC profile was defined as the row of the structural connectivity matrix representing tractography-based streamline counts between a specific region and all other regions. The FC profile was defined as the corresponding row of the functional connectivity matrix representing Pearson correlation coefficients. For each region, we calculated the Spearman rank correlation between its structural and functional profiles (excluding self-connections). These regional coupling coefficients were then related to the neuropsychological measures using the same regularized Canonical Correlation Analysis (CCA) pipeline as in Fig. 5 main text.

E.2 Quantitative Comparison

We compared whether our proposed gap measures based on prediction residuals had more power in identifying meaningful brain-behavior relationships than the traditional SC-FC coupling metrics. Given the methodological difference between the two approaches, a direct power comparison is not straightforward. We therefore conducted a controlled comparison by matching the input dimensionality of the coupling measures used for downstream CCA analyses.

Because our proposed gap measures were 25-dimensional (corresponding to the number of major white-matter tracts with significant prediction accuracy), we derived traditional SC-FC coupling measures in two ways. First, we randomly selected 25 cortical regions from the SRI24 atlas. For each participant, we computed the Spearman correlation between the SC and FC profiles of each selected region, yielding 25 regional coupling measures to match the dimensionality of our gap measures. In doing so, the canonical correlation of the first CCA component was consistently lower than that obtained using the gap measures (mean ± SD: 0.06 ± 0.03), indicating that given the same input dimensionality, the gap measures based on prediction residuals provided greater statistical power for capturing brain-behavior relationships.

Second, when using all 40 cortical regions to compute traditional SC-FC coupling mea-

sures, the resulting CCA achieved canonical correlations comparable in magnitude to those obtained with our gap measures (first component $r = 0.10$; second component $r = 0.08$, Fig. 5a). Importantly, this CCA identified 20 neuropsychological variables with significant loadings, and 14 out of the 20 variables (70%) were also captured by the CCA model based on the proposed gap measures (Table A4, Figure A7), indicating substantial convergence between the two models. Although the identified brain regions were not directly comparable between the two models (one approach operated on white-matter tracts, and the other approach operated on cortical regions), the dominant neurobiological circuits were highly consistent. One component maps to the Fronto-Limbic regulatory system, while the other converges on the Limbic-Cingulate circuit, supporting the biological reliability and interpretability of the structure-function relationships identified by our framework.

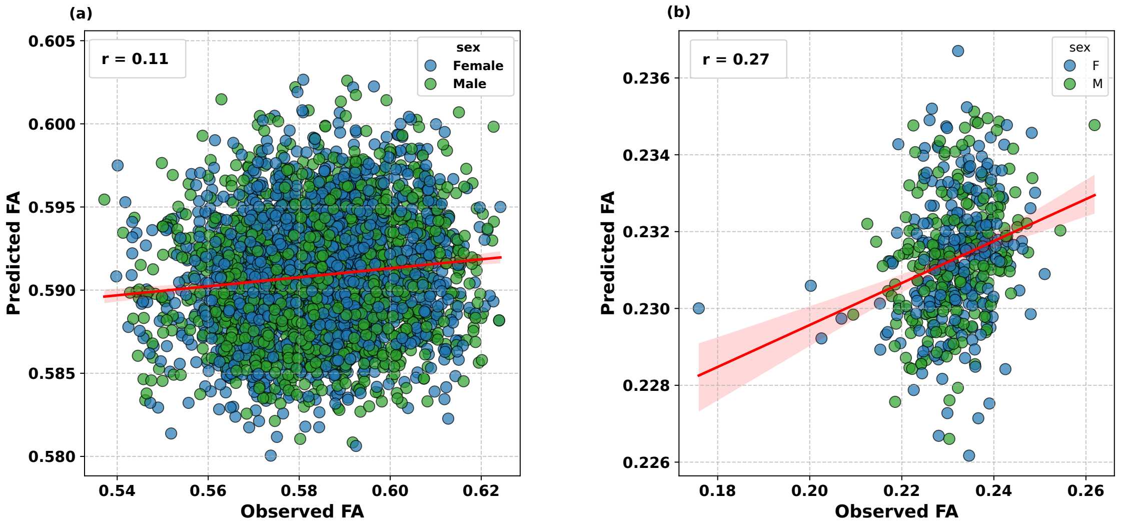

Figure A1: (a) Cross-validating the GP model on NCANDA data using FC among 106 regions revealed that the predicted whole-brain FA significantly correlated with the observed FA in DTI; (b) GP prediction of whole-brain FA from FC in the HCP-D dataset. Using 5-fold cross-validation, the model achieved $r = 0.27$.

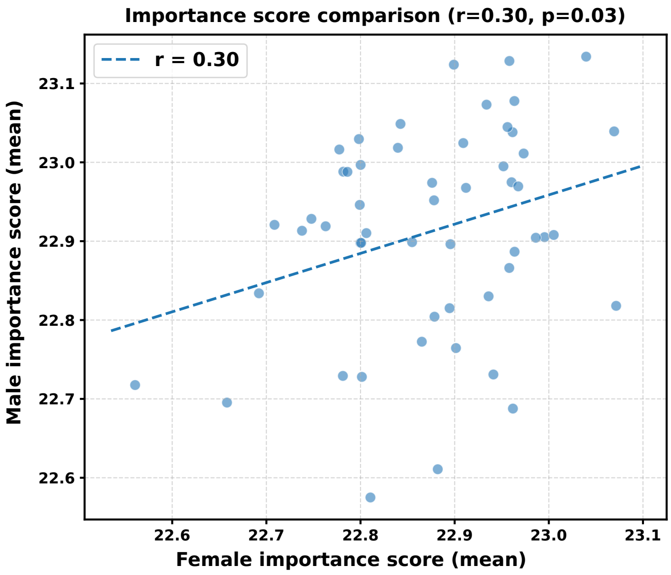

Figure A2: Cross-sex stability of importance scores obtained from Whole-brain FA: The importance scores derived from a model trained exclusively on female subjects (x-axis) significantly correlated with the importance scores derived from a model trained exclusively on male subjects (y-axis).

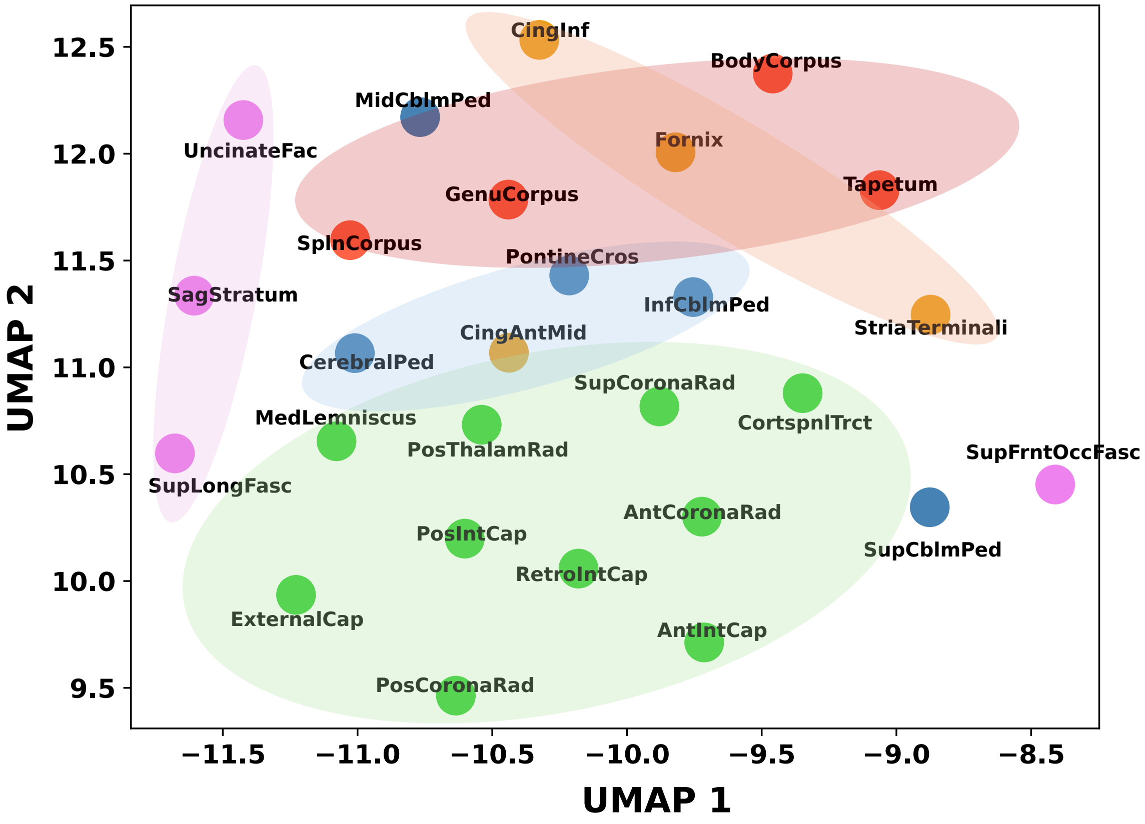

Figure A3: 2D UMAP visualization of the model length-scale associated with all 27 white-matter regions indicated that regions belonging to the same fiber tract significantly clustered together ($p < 0.01$, PERMANOVA).

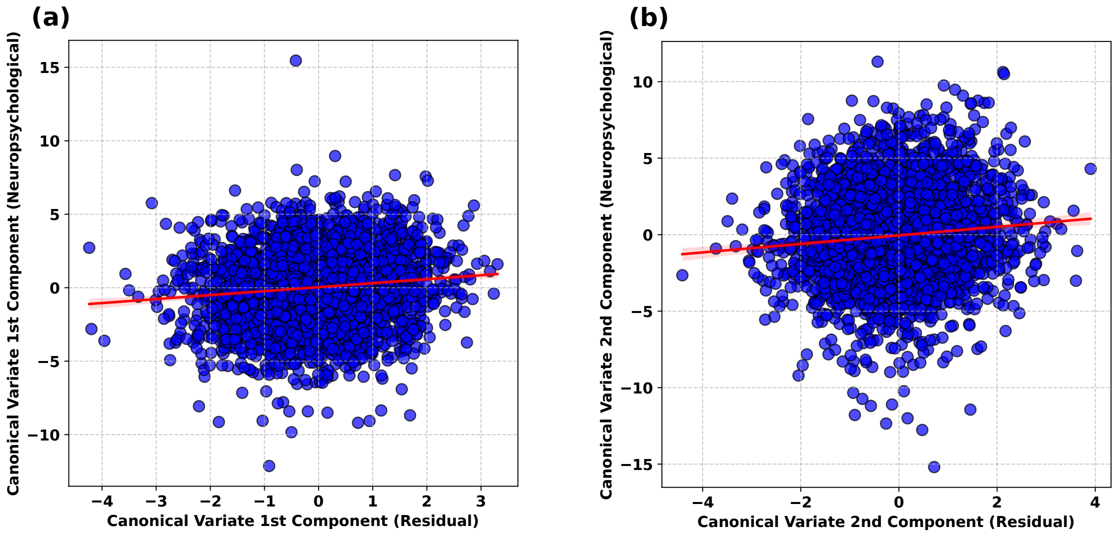

Figure A4: Scatter plots for (a) the 1st component and (b) the 2nd component of the leading canonical covariates in Fig. 5a in the main text.

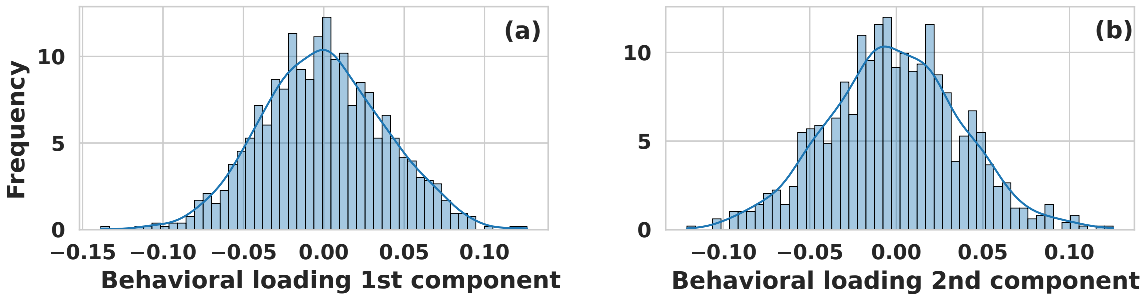

Figure A5: Null distributions of (a) behavioral loadings for 1st component, (b) and behavioral loadings for the 2nd component

References

Baum, G. L., Cui, Z., Roalf, D. R., Ciric, R., Betzel, R. F., Larsen, B., Cieslak, M., Cook, P. A., Xia, C. H., Moore, T. M., et al. (2020). Development of structure–function coupling in human brain networks during youth. *Proceedings of the National Academy of Sciences*, *117*(1), 771–778.

Gu, Z., Jamison, K. W., Sabuncu, M. R., & Kuceyeski, A. (2021). Heritability and interindividual variability of regional structure-function coupling. *Nature Communications*, *12*(1), 4894.

Table A1: Mapping of white-matter region abbreviations to full anatomical names.

**Tract**	**Abbreviation**	**27 white-matter Regions Defined by the JHU-Mori Atlas**
Commissural	GenuCorpus<br>BodyCorpus<br>SplnCorpus<br>Tapetum	Genu of Corpus Callosum<br>Body of Corpus Callosum<br>Splenium of Corpus Callosum<br>Tapetum
Brainstem	MidCblmPed<br>PontineCros<br>InfCblmPed<br>SupCblmPed<br>CerebralPed	Middle Cerebellar Peduncle<br>Pontine Crossing Tract<br>Inferior Cerebellar Peduncle<br>Superior Cerebellar Peduncle<br>Cerebral Peduncle
Limbic	Fornix<br>CingAntMid<br>CingInf<br>StriaTerminali	Fornix<br>Cingulum (Cingulate Gyrus) - Anterior<br>Cingulum (Hippocampal) - Inferior<br>Stria Terminalis
Fasciculi	SagStratum<br>SupLongFasc<br>SupFrntOccFasc<br>UncinateFac	Sagittal Stratum<br>Superior Longitudinal Fasciculus<br>Superior Fronto-Occipital Fasciculus<br>Uncinate Fasciculus
Corticospinal	CortspnlTrct<br>MedLemniscus<br>AntIntCap<br>PosIntCap<br>RetroIntCap<br>AntCoronaRad<br>SupCoronaRad<br>PosCoronaRad<br>PosThalamRad<br>ExternalCap	Corticospinal Tract<br>Medial Lemniscus<br>Anterior Limb of Internal Capsule<br>Posterior Limb of Internal Capsule<br>Retrolenticular Part of Internal Capsule<br>Anterior Corona Radiata<br>Superior Corona Radiata<br>Posterior Corona Radiata<br>Posterior Thalamic Radiation<br>External Capsule

Vázquez-Rodríguez, B., Suárez, L. E., Markello, R. D., Shafiei, G., Paquola, C., Hagmann, P., Van Den Heuvel, M. P., Bernhardt, B. C., Spreng, R. N., & Misic, B. (2019). Gradients of structure–function tethering across neocortex. *Proceedings of the National Academy of Sciences*, *116*(42), 21219–21227.

Table A2: Stability of Gaussian process lengthscales across 5-Fold cross-validation. The Coefficient of Variation (CoV) (%) represents the stability of feature weights for a prediction model. A lower percentage indicates higher consistency of the selected features and their weights across the folds.

**Target Region**	**Mean CoV(%)**	**Target Region**	**Mean CoV(%)**
global_mori	1.61	RetroIntCap	1.55
MidCblmPed	1.43	AntCoronaRad	1.97
PontineCros	0.71	SupCoronaRad	1.12
GenuCorpus	1.09	PosCoronaRad	1.07
BodyCorpus	1.54	PosThalamRad	1.17
SplnCorpus	1.29	SagStratum	0.82
Fornix	1.51	ExternalCap	0.90
CortspnlTrct	0.83	CingAntMid	1.07
MedLemniscus	1.80	CingInf	1.27
InfCblmPed	1.44	StriaTerminali	0.93
SupCblmPed	1.70	SupLongFasc	0.81
CerebralPed	1.62	SupFrntOccFasc	1.52
AntIntCap	1.59	UncinateFac	0.93
PosIntCap	2.12	Tapetum	0.83

Note: Coefficient of variation was calculated as the standard deviation of lengthscales across 5 folds divided by the mean coefficient magnitude, averaged across all 1,431 input features.

Table A3: Gray-matter brain regions defined in the SRI24 atlas grouped by their functional networks.

**Network Name**	**Region Name**
Visual Network (VN)	Calcarine
	Cuneus
	Lingual
	Occipital_Sup
	Occipital_Mid
	Occipital_Inf
	Fusiform
Somatomotor Network (SMN)	Precentral
	Rolandic_Oper
	Supp_Motor_Area
	Postcentral
	Heschl
	Temporal_Sup
	Paracentral_Lobule
	Temporal_Inf
	SupraMarginal
Dorsal Attention Network (DAN)	Parietal_Sup
	Parietal_Inf
	Temporal_Inf
	Fusiform
Ventral Attention Network (VAN)	SupraMarginal
	Parietal_Sup
	Parietal_Inf
	Temporal_Inf
Limbic Network (LN)	Frontal_Inf_Oper
	Insula
	Cingulum_Mid
	SupraMarginal
	Cingulum_Ant
	Rectus
Frontoparietal Network (FPN)	Frontal_Sup_Orb
	Olfactory
	Rectus
	Hippocampus
	ParaHippocampal
	Amygdala
	Caudate
	Putamen
	Thalamus
Default Mode Network (DMN)	Frontal_Sup
	Frontal_Mid
	Frontal_Mid_Orb
	Frontal_Inf_Tri
	Frontal_Inf_Orb
	Frontal_Sup_Medial
	Frontal_Med_Orb
	Cingulum_Ant
	Cingulum_Post
	Angular
	Precuneus
	Temporal_Pole_Sup
	Temporal_Mid
	Temporal_Pole_Mid
Cerebellum (CB)	Cerebellum_Crus1
	Cerebellum_Crus2
	Cerebellum_3
	Cerebellum_4_5
	Cerebellum_6
	Cerebellum_7b
	Cerebellum_8
	Cerebellum_9
	Cerebellum_10

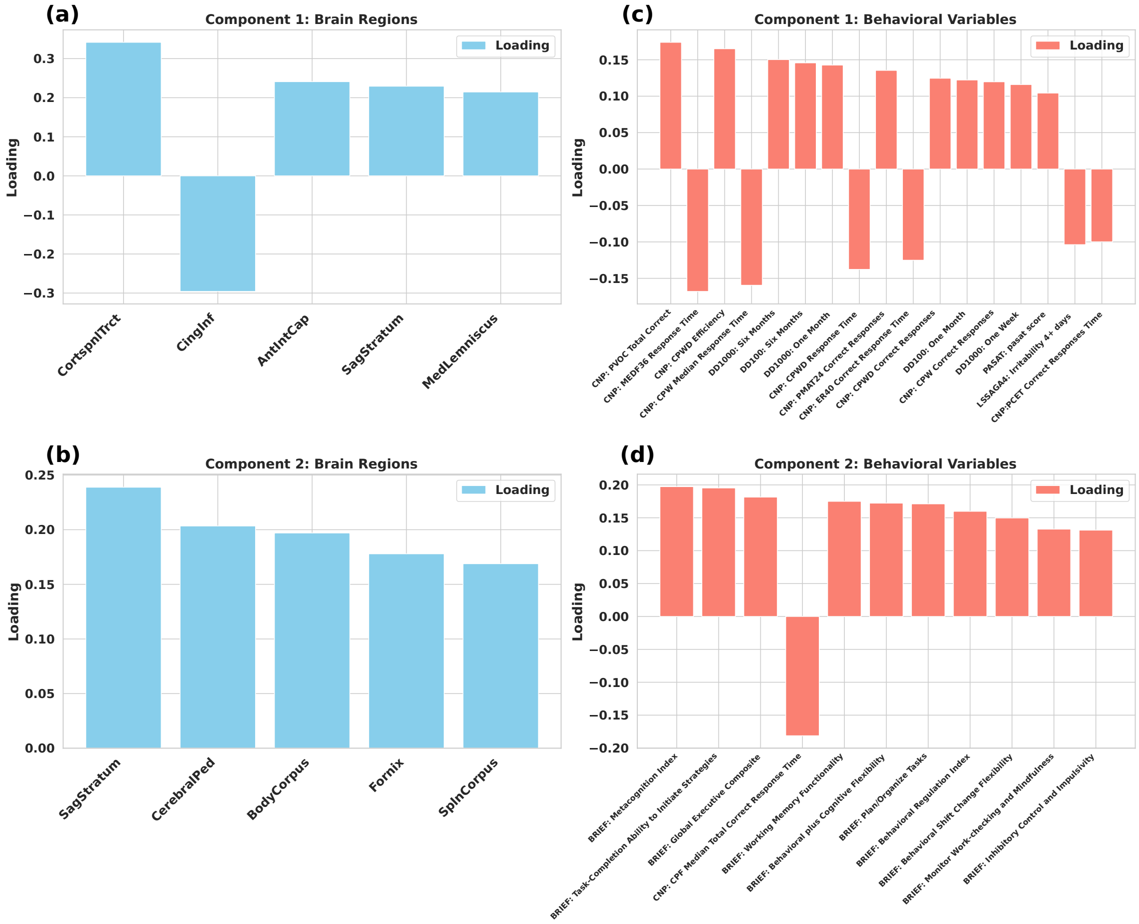

Figure A6: Signed loadings associated the brain regions and neuropsychological measures identified in the two significant CCA components in Main Text Fig. 5.

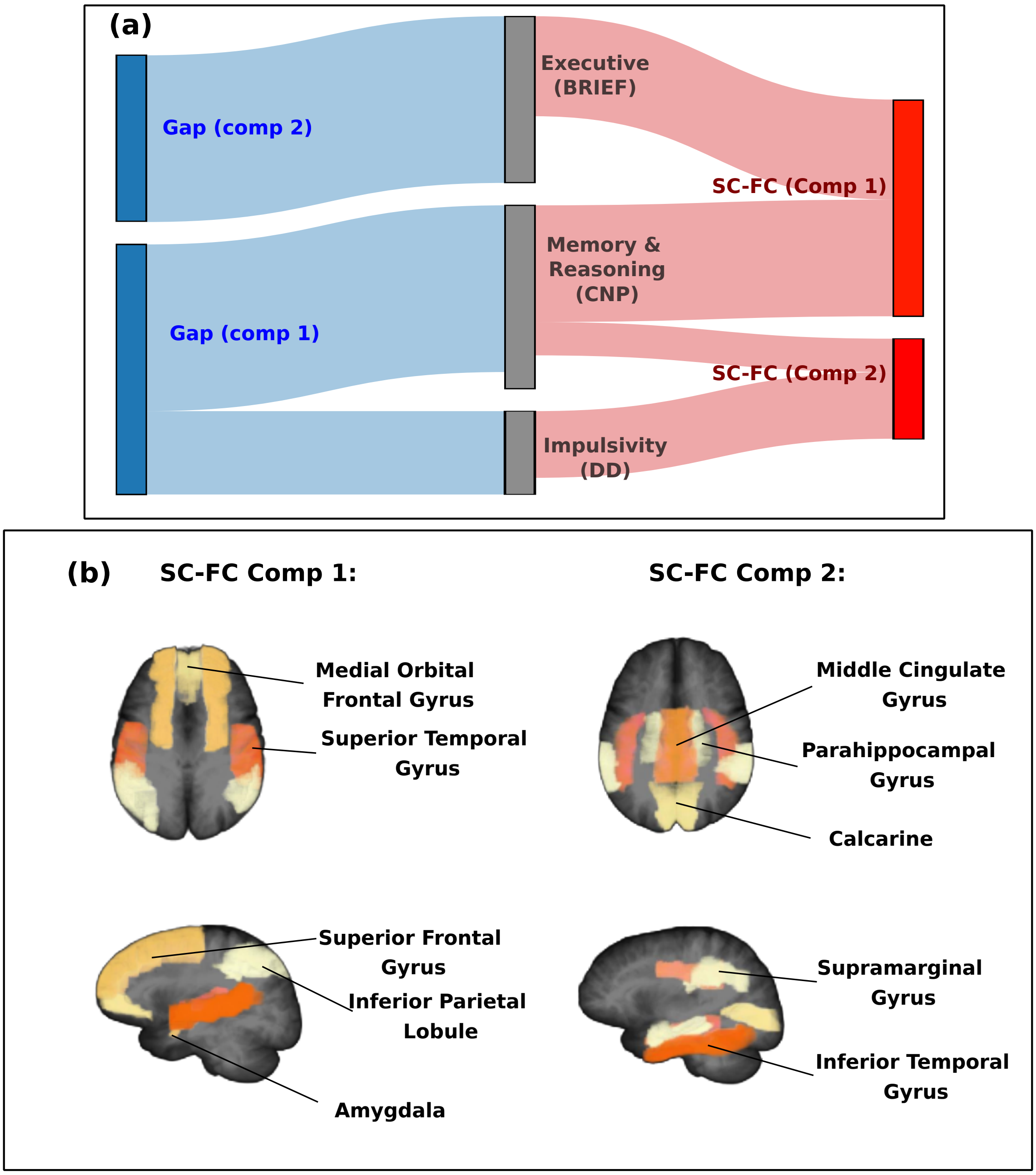

Figure A7: Comparing identified neuropsychological and brain variables with high loadings between the two CCA models based on the proposed Structure-Function Gap measures or the traditional SC-FC Coupling metrics: (a) 14 neuropsychological variables were identified in both CCA models in the first two canonical components (Table A4); (b) Top 5 cortical regions with highest loadings in the first two components identified by the CCA model using traditional SC-FC coupling metrics.

Table A4: Detailed comparison of common neuropsychological variables identified by the two CCA models based on the proposed Structure-Function Gap measures or traditional SC-FC Coupling metrics.

**Neuropsychological Measure**	**Proposed Gap (Component)**	**SC-FC Coupling (Component)**
Executive Function (BRIEF)		
Metacognition Index	**2**	1
Task-Completion Ability to Initiate Strategies	**2**	1
Global Executive Composite	**2**	1
Plan/Organize Tasks	**2**	1
Inhibitory Control and Impulsivity	**2**	1
Working Memory Functionality	**2**	1
Impulsivity (Delay Discounting)		
DD1000: Six Months	**1**	2
DD1000: One Month	**1**	2
DD1000: One Week	**1**	2
Cognition & Memory (CNP/PASAT/LSSAGA)		
CPF Median Total Correct Response Time	**2**	1
MEDF36 Response Time	**1**	1
CPW Median Response Time	**1**	1
CPWD Response Time	**1**	1
PMAT24 Correct Responses	**1**	2
